# The 4-Hydroxynonenal–Protein Adducts and Their Biological Relevance: Are Some Proteins Preferred Targets?

**DOI:** 10.3390/antiox12040856

**Published:** 2023-04-01

**Authors:** Lidija Milkovic, Neven Zarkovic, Zlatko Marusic, Kamelija Zarkovic, Morana Jaganjac

**Affiliations:** 1 Laboratory for Oxidative Stress, Division of Molecular Medicine, Ruder Boskovic Institute, Bijenicka 54, 10000 Zagreb, Croatia; 2 Division of Pathology, Clinical Hospital Centre Zagreb, Kispaticeva 12, 10000 Zagreb, Croatia

**Keywords:** lipid peroxidation, 4-hydroxynonenal (4-HNE), 4-HNE–protein adducts, immunochemical methods, mass spectrometry (MS), adaptive response, the NRF2/KEAP1 signaling, ferroptosis

## Abstract

It is well known that oxidative stress and lipid peroxidation (LPO) play a role in physiology and pathology. The most studied LPO product with pleiotropic capabilities is 4-hydroxynonenal (4-HNE). It is considered as an important mediator of cellular signaling processes and a second messenger of reactive oxygen species. The effects of 4-HNE are mainly attributed to its adduction with proteins. Whereas the Michael adducts thus formed are preferred in an order of potency of cysteine > histidine > lysine over Schiff base formation, it is not known which proteins are the preferred targets for 4-HNE under what physiological or pathological conditions. In this review, we briefly discuss the methods used to identify 4-HNE–protein adducts, the progress of mass spectrometry in deciphering the specific protein targets, and their biological relevance, focusing on the role of 4-HNE protein adducts in the adaptive response through modulation of the NRF2/KEAP1 pathway and ferroptosis.

## 1. Introduction

As aerobic beings, we need oxygen for our energy metabolism and physiological processes. Consequently, a small amount of oxygen is converted into reactive oxygen species (ROS). Their increased formation exceeds the level that the antioxidant machinery can cope with and is referred to as oxidative stress [1]. ROS were first perceived as detrimental, with oxidative stress leading to various diseases such as cancer, neurodegenerative disorders, autoimmune diseases, cardiovascular diseases, etc., [2,3]. However, further research has revealed their concentration-dependent role, highlighting them also as regulators of redox-sensitive signaling pathways and thus of various cellular processes [4]. Nowadays, we distinguish between eustress (the good one) and distress (the bad one) [5]. As recently pointed out by experts, ROS differ in their signaling abilities and effects on the cell, which requires a specific interpretation of their role. To be precise, among ROS, the signaling abilities are mainly attributed to hydrogen peroxide and to a lesser extent to superoxide anion. Alternatively, the highly reactive hydroxyl radical is considered detrimental. It readily and non-specifically reacts with cellular macromolecules (proteins, nucleic acids, lipids), the products of which can be determined as potential biomarkers of oxidative stress [6]. Studying how ROS affect cellular processes is challenging due to their hectic nature. As mentioned earlier, other biomarkers aided in understanding the involvement of oxidative stress in diverse pathophysiological processes and diseases.

Lipid peroxidation (LPO) is considered the accompanying event of oxidative stress. It is an autocatalytic chain reaction initiated by free radical attack (e.g., hydroxyl radical, hydroperoxyl radical) on the carbon–carbon double bonds of polyunsaturated fatty acids (PUFA). LPO can be triggered enzymatically. Various products are formed during LPO [7]. Lipid hydroperoxides occur primarily, whereas further oxidation leads to the formation of secondary end products such as reactive aldehydes [8,9]. 4-Hydroxynonenal (4-HNE) is the most studied and seemingly the most biologically relevant product of LPO.

**Figure 1:**
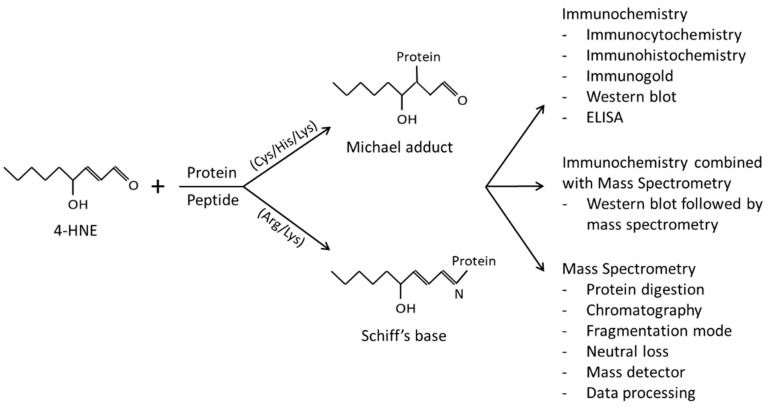
4-HNE forms adducts with protein amino acid residues via Michael addition or Schiff’s base formation that are frequently analyzed by immunochemistry, mass spectrometry, or a combination of immunochemistry with mass spectrometry.

4-HNE is an α,β-unsaturated aldehyde derived from arachidonic or linoleic acid either by their oxidation due to free radical attack (reviewed by Prof. Esterbauer and colleagues in [8]) or enzymatically by lipoxygenases [10,11,12]. Another suggested route for the formation of 4-HNE involves the oxidation of cardiolipin, a linoleic-acid-rich phospholipid found predominantly in mitochondria [13]. Cells readily metabolize 4-HNE. Its detoxification involves conjugation with glutathione catalyzed by glutathione S-transferase and oxidation or reduction by aldehyde dehydrogenase or alcohol dehydrogenase, respectively. Metabolic elimination is cell/tissue-type specific [14]. It is estimated that about 2–8% of 4-HNE remains free to react with proteins to form 4-HNE–protein adducts [15]. Three functional groups (double bond, carbonyl group, and hydroxyl group) contribute to the electrophilic nature and high reactivity of 4-HNE. It reacts rapidly, mainly with cysteine (Cys), histidine (His), lysine (Lys), and to some extent with arginine (Arg) residues of proteins, forming Michael adducts or Schiff’s bases (Figure 1). The predominant reaction is the formation of Michael adducts in the order of reactivity Cys > His > Lys > Arg [16,17,18]. The kinetic studies on the rate of formation of 4-HNE adducts with nucleophilic amino acids show a pH dependence and a preference for Cys thiolate sites over His, Lys, or thiol residues [19]. A preference for Cys was also found for other α,β-unsaturated aldehydes such as acrolein and 4-oxononenal (4-ONE) but with different reactivity and depending on the protein structure and Cys pK_a_ [17,20]. The kinetic assessment of Michael adduct formation has been described in great detail in several excellent papers [17,20]. In addition to the preferred amino acids mentioned above, modification of lysozyme and bovine serum albumin with 4-HNE was found to result in the formation of a Michael adduct at the threonine (Thr) residue, the formation of Schiff’s base at the tryptophan (Trp) residue, and the formation of a pyrrole-type adduct with His, which was previously known only for the Lys residue [21]. In addition, Annangudi et al. have shown that more molecules of 4-HNE (2:1 and 3:1) might be adducted to histidyl or lysyl nucleophiles [22]. Stereoselectivity also contributes to the formation of 4-HNE–protein adducts. For thioredoxin, the predominant modification with 4-HNE is Cys73 and to a lesser extent is Cys32. Although both forms, (*R*)-HNE and (*S*)-HNE, equally form adducts with Cys73, the modification of Cys32 is mainly with (*R*)-HNE [23].

Some argue that LPO products could not be signaling molecules due to their diversity, the formation of which appears to lack precise regulation [24]. Moreover, 4-HNE modifications of proteins mainly lack a reversible modification—“switch on and off”—to be considered as a true signaling molecule, although some, in particular Cys modifications, could be reversed to some extent in the presence of glutathione [25,26]. However, the observed pleiotropic and concentration-dependent effects of 4-HNE on cellular processes [27] in both physiology and pathology [28] challenge this opinion. Indeed, emerging evidence for the selectivity of 4-HNE–protein adduction sites favors the perception of 4-HNE being an important mediator of signaling processes, as nicely reviewed by Zhang and Forman [29]. Its signaling abilities are rather attributed to 4-HNE–protein adducts than to free 4-HNE [30].

In this review, we will mention methods used to study 4-HNE–protein adducts, including immunochemistry and mass spectrometry (Figure 1), and the biological relevance of the specific 4-HNE–protein adducts.

## 2. Immunochemical Methods Employed for the Detection of 4-HNE–Protein Adducts

Only with the development of antibodies specific for 4-HNE–protein adducts has the research of their involvement in stress-related processes/diseases begun. The evolution of various 4-HNE-raised antibodies started with the development of polyvalent antisera and a monoclonal antibody against cyanoborohydride-reduced 4-HNE-treated low-density lipoprotein (LDL) [31] or 4-HNE–LDL [32]. Palinski et al. [31] reported that a 4-HNE–LDL adduct prepared under reducing conditions is a better immunogen than the non-reduced one. They used this reduced 4-HNE–LDL and developed a polyvalent antiserum and a monoclonal antibody that they reported to specifically recognize a 4-HNE–Lys epitope on LDL but also on other proteins, whereas being unreactive with native LDL or malondialdehyde (MDA)–LDL. At the same time, Prof. Esterbauer’s group developed an antiserum against unreduced 4-HNE–LDL, which did not react with LDL treated with hexanal or hepta-2,4-dienal or 4-hydroxyhexenal or MDA but slightly reacted with 4-hydroxyoctenal–LDL. The antiserum also reacted with cooper-oxidized LDL, oxidized lipoprotein (a), and very low density lipoprotein [32]. Although Palinski et al. reported that antibodies are specific for the 4-HNE–Lys epitope, further research by Prof. Esterbauer’s group showed that the antiserum recognizes not only 4-HNE–Lys but also 4-HNE–Tyr, 4-HNE–Arg, and 4-HNE–His epitopes [33]. Similarly, Uchida et al. developed a polyclonal antibody against 4-HNE–keyhole limpet hemocyanin (KLH), which also reacted with HNE–LDL and cooper-oxidized LDL [34]. The antiserum was purified on a 4-HNE–histidyl-peptide column by affinity chromatography, and they showed it to be reactive with all Michael-type 4-HNE adducts, including 4-HNE–His, 4-HNE–Lys, and 4-HNE–Cys [35]. The aforementioned research suggested that the 4-HNE moiety, regardless of the 4-HNE–amino acid conjugate, is responsible for the specificity of the mentioned antibodies. In addition, while investigating which are the predominant epitopes recognized by the antiserum, Prof. Esterbauers’s group also found that another batch of antiserum prepared for this research showed some reactivity with hexanal–LDL and 2,4-heptadienal–LDL, which was not observed in the first one [33]. This illustrates the natural variation that is present in polyclonal preparations and led to the development of monoclonal antibodies with higher specificity.

Almost in parallel two groups developed the monoclonal 4-HNE antibodies [36,37]. Both used 4-HNE–KLH as an immunogen and 4-HNE-treated bovine serum albumin (BSA) for screening the most specific and reactive clones. Toyokuni et al. stated the HNEJ-2 clone did not react with 2-nonenal, 2-hexenal, 1-hexanal, 4-hydroxyhexenal, formaldehyde, or glutaraldehyde and is specific to the 4-HNE–His epitope [36], thus supporting their previous research suggesting the 4-HNE–histidine Michael addition reaction is biologically important [38]. Likewise, Waeg et al. selected a 1g4 clone that did not cross-react with proteins modified by malonaldehyde, nonanal, nonenal, and 4-hydroxyhexenal or negligibly with 4-hydroxyoctenal, and 4-hydroxydecenal. Additionally, this antibody was highly selective for the 4-HNE–His epitope and used to quantify the amount of 4-HNE–His by ELISA in oxidized microsomes and oxidized LDL revealing they contain 12 nmol and 3 nmol 4-HNE–His/mg protein, respectively [37]. Capping of His of the BSA supported the Ig4 antibody to be specific for the 4-HNE–His epitope while at the same time revealing relatively high recognition of other 4-HNE–amino acid epitopes for HNEJ-2 [39].

In addition, Hartley and colleagues developed rabbit polyclonal antibodies raised to be selective for 4-HNE–Cys for studying the involvement of 4-HNE–protein adducts in liver prooxidant-induced toxicity [40]. They used 4-HNE–glutathione (GSH) hapten crosslinked with KLH for immunization and CCl_4_ or iron/ascorbate to induce oxidative stress in hepatocytes. Neither 4-HNE nor 4-HNE–protein adducts changed upon treatments, whereas MDA and MDA-modified proteins did [40]. Further attempts to develop a specific antibody against 4-HNE-modified proteins that could be used in human diagnostics led to the consideration of using a different immunogen. Recently, Prof. Spickett’s group developed a polyclonal antibody against 4-HNE-modified human serum albumin (HSA), characterized it by high-resolution mass spectrometry (MS), and compared it with the commercial polyclonal antibody raised against HNE–KLH for the investigation of the epitope recognition. High-resolution MS confirmed 15 unique HNE-adduct-containing peptides in HNE-treated HSA. Cys34, the only free thiol, was carbamidomethylated but not modified by HNE. Reversible modifications such as cysteinylation may have prevented further HNE modification but cannot be ruled out because of the reduction–alkylation protocol used. Most of the epitopes detected by both antibodies contained His, with the highest affinity for peptide ^365^DPHECYAKVFDEFKPLV^381^, which contains the potential target amino acids His367, Lys372, and Lys378 [41].

Although a variety of polyclonal 4-HNE antibodies is commercially available, they are often not well characterized for their specificity and selectivity. Table 1. contains a list of commercially available clones of which the HNEJ-2 is mainly used and the best characterized.

**Figure 2:**
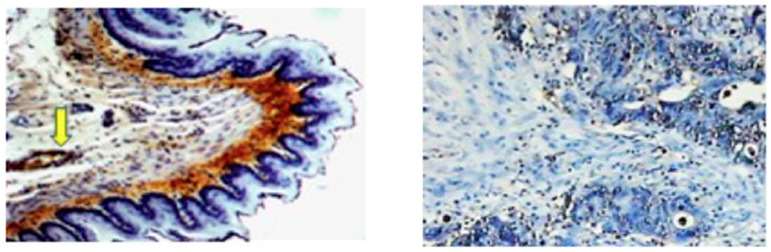
Immunohistochemistry with the monoclonal antibody specific for the 4-HNE–His shows the prominent presence of the protein adducts of the aldehyde in connective tissue (brown) below the 4-HNE–His negative epithelium (blue) of esophagus of a healthy rat (**left** photo 100×) and in the blood vessel (indicated by the yellow arrow). In human colon cancer, 4-HNE can be seen only in the nuclear region of a few cancer cells (**right** photo 50×). In both cases the presence of 4-HNE–protein adducts is visualized by dark brown di-amino-benzidine staining, with the blue hematoxylin contrast staining.

Despite the limitations of the antibody approach, immunochemical methods allowed a better understanding of the tissue [42] and subcellular distribution of 4-HNE–protein adducts using immunoelectron microscopy, which revealed that both endogenous and exogenously added 4-HNE mainly affects the protein residues of cellular membrane structures [43]. In addition, immunochemistry helped to quantify and compare the appearance of 4-HNE–protein adducts depending on the condition/disease [39,44] (Figure 2) and to reveal which proteins are modified by 4-HNE [45]. All this work has increased our knowledge of the impact that 4-HNE–protein adducts have in health and disease [46], whereas the ELISA specific for 4-HNE–His adducts was found to be comparable with the “golden standard” method, HPLC-MDA, for measuring LPO products in UV-irradiated human plasma [47]. However, 4-HNE–protein specificity and the underlying mechanisms should be further explored.

## 3. 4-HNE Adduction to Proteins—Selective or Random Event?

Whether 4-HNE randomly or selectively modifies proteins has been a matter of debate for more than two decades. Based on the chemistry and reactivity of 4-HNE, one could already advocate the 4-HNE selectivity for proteins containing Cys, His, or Lys residues because the probability for proteins that contain other amino acids to be modified with 4-HNE is low. However, despite kinetic data showing that modification of Cys is strongly favored over reaction with Lys, His, and Arg, this preference may vary depending on other factors such as protein structure and residue location. For example, in α-synuclein the main target for 4-HNE adduction is His50, promoting α-synuclein oligomerization [48], whereas the major target of 4-HNE on cytochrome C was reported to be His33 [49]. In addition, quantitative chemoproteomics identified the CxxxK motif characteristic for 4-HNE protein alkylation [50]. The 4-HNE preference for different amino acids, specific sequence motifs, or amino acid location within the protein was discussed in an excellent review by Zhang and Forman [29].

Tzeng and colleagues have shown that the susceptibility of proteins to 4-HNE adduction is highly variable. They found that 4-HNE has different reactivity profiles for adduct formation depending on its concentration [51]. The same study identified 31 protein targets of 4-HNE, which according to the reactivity profile dependent on 4-HNE concentration, might suggest that the 4-HNE adductome selectively affects different processes depending on the degree of lipid peroxidation [51]. In vitro studies on the 4-HNE exposure of erythrocyte membranes reported selective 4-HNE modification of spectrin proteins [52]. There are a number of proteins, such as enolase and ATP synthase alpha, whose selective modification with 4-HNE has been associated with the Alzheimer’s disease progression [53].

A recent study investigated the presence of MDA–Lys, 4-HNE–Cys, 4-HNE–Lys and 4-HNE–His adducts in keratinocytes under normal and stress conditions [54]. After UVB irradiation, alpha-enolase, annexin, cadherin-12, G6PD, glutathione S-transferase, HSP90, proteasome subunits, and tubulin were among proteins modified only with 4-HNE. Contrarily, synaptotagmin-like protein 2 was modified only with MDA [54]. A study by Just and colleagues identified 4-HNE adducts on 20S proteasomal subunit α7 that were unstable and reversible, suggesting a possible regulatory role of 4-HNE adducts on proteasomal activity [55]. Furthermore, a comparative study of ubiquitin modification with 4-HNE, methylglyoxal, glyoxal, and MDA revealed the highest reactivity for methylglyoxal [56]. However, each reactive carbonyl demonstrated a different preferential site for modification. The preferential target for 4-HNE was His68 and it was Arg74 for methylglyoxal, Arg42 for glyoxal, and Lys29 for MDA [56]. Comparison of the effects of 4-HNE and n-3 PUFA peroxidation product 4-hydroxy-2-hexenal (HHE) revealed a higher reactivity of 4-HNE towards adduct formation with insulin, thus reducing insulin-induced glucose uptake [57]. In a mouse diet-induced obesity model, it was found that 4-HNE and 4-HHE have different preferences for adipocyte nuclear proteins [58]. Both aldehydes had a similar preference for Cys; however, their preference varied for Lys and His modifications. Moreover, although a higher number of modified peptides was recorded for 4-HHE, they corresponded to a lower number of proteins compared with 4-HNE-modified proteins [58].

Exposure of human glucose regulated protein 78 (GRP78) to physiological 4-HNE and 4-ONE concentrations revealed a greater number of amino acids in the ATPase region prone to 4-HNE modification compared with 4-ONE, whereas His477 in the peptide-binding region was found to be adducted only with 4-HNE [59].

It is thus clear that protein interactions with 4-HNE are frequently distinct from interactions with other reactive aldehydes. This could be in part attributed to the oxidation products will deffer between for example peroxidation of omega-3 or omega-6 PUFAs as well as to the 4-HNE reactivity and target preference.

## 4. Mass Spectrometry as a Tool to Study the 4-HNE–Protein Adductome

Technical breakthroughs in mass spectrometry technologies have positioned mass spectrometry as an essential tool for proteomics research. In addition to protein identification and quantification, mass spectrometry is also essential for the analysis of posttranslational modifications, such as protein modifications with 4-HNE. Identification of specific amino acid residues modified by 4-HNE contributes to our understanding of the underlying mechanisms of various physiological and pathological processes. Due to the ability of 4-HNE to form adducts with proteins [18], Cys, His, Lys, and Arg residues are commonly analyzed by mass spectrometry for the presence of 4-HNE modifications. Recognition of 4-HNE as an important bioactive molecule and a modulator of cellular processes in physiology and pathology [7,28,60] has highlighted the importance of studying the 4-HNE protein adductome. Early studies used immunochemistry to detect stable 4-HNE-adducts followed by protein identification by mass spectrometry using electrospray ionization (ESI) or matrix-assisted laser desorption/ionization (MALDI) as ionization methods. The change in the mass to charge ratio of a particular peptide observed by mass spectrometry is indicative of adduct formation. Depending on Michael addition or Schiff base formation as well as the peptide charge, different mass shifts for a peptide can be expected. Table 2 shows the variety of methodological approaches used for 4-HNE adductome analysis in complex mixtures, from the sample preparation techniques themselves to the mass analyzers used.

With the development of technologies and in order to reduce the time and effort required for protein identification using 4-HNE immunostaining, the analysis of site-specific 4-HNE protein modifications directly by mass spectrometry coupled to liquid chromatography is receiving more and more attention. This is particularly relevant for complex matrices, and today mass spectrometry analysis of the 4-HNE adductome by liquid chromatography tandem mass spectrometry (LC-MS/MS) is an indispensable tool. Despite the sensitivity and accuracy of the available mass spectrometry methods, the identification of low abundance proteins is analytically challenging. To address this issue, samples can be enriched for the organelle, protein, or modification of interest. Different approaches are used to identify 4-HNE protein targets regardless of the chemical bond between them. Some approaches aim to detect all proteins modified with 4-HNE by the immunoprecipitation of 4-HNE-modified proteins using labeling with biotin hydrazide or click chemistry [63,66,67,73,74,75], whereas other approaches are organelle oriented or protein specific and enrich for the proteins of interest [58,76].

## 5. Biological Relevance of Protein Residue Modification by 4-HNE

The bioactive function of 4-HNE is nowadays well recognized and a number of proteins have been found to be susceptible to 4-HNE modification in both physiology and pathology, including aging [77]. For example, oscillations of cytosolic calcium, which are required for muscle contraction and for calcium-dependent upregulation of mitochondrial metabolism, are accompanied by low rates of lipid peroxidation and formation of 4-HNE adducts that regulate mitochondrial metabolism in skeletal myotubes [78]. In contrast, elevated 4-HNE reduces mitochondrial creatinine kinase activity and leads to structural changes [79]. During aging, 4-HNE protein adducts accumulate in the tissues. Co-localization of 4-HNE adducts with epidermal growth factor receptor is associated with the loss of elastin [80], whereas 4-HNE adduction to proteasomal units leads to impaired protein homeostasis in aged cells [81]. Gap junction Cx46 hemichannels are also sensitive to 4-HNE and under high 4-HNE concentrations are carbonylated with 4-HNE, impairing their function [82]. The involvement of 4-HNE protein adducts has been implicated in a number of pathological conditions including metabolic syndrome and cancer. In obesity, 4-HNE protein adducts accumulate in both subcutaneous and omental adipose tissue, where 4-HNE has been shown to impair adipogenesis and induce insulin resistance [83,84]. In tumorigenesis, the accumulation of 4-HNE–protein is tumor specific, and whereas in some cancer types 4-HNE–protein adducts accumulate predominantly in tumor cells compared with the surrounding stroma, the opposite results have been observed in other types, as reviewed recently [7,28]. In the recent COVID-19 pandemic, the accumulation of HNE–protein adducts was associated with the severity and lethal outcome of SARS-CoV-2 infection, not due to specific pneumonia but due to systemic oxidative and vascular stress based on the penetration of 4-HNE from the blood into the tissues of vital organs [85,86,87].

Immunochemical detection of 4-HNE–protein adducts in COVID-19 patients failed to reveal the pathogenic mechanisms of the observed changes, although it has been suggested that vascular oxidative stress may be crucial. This was also previously suggested to be crucial for the systemic effects of 4-HNE–protein adducts by the analysis of an animal model of atherosclerosis [88], by immunohistochemistry of the atherosclerotic human aorta [89], and more recently by comparing the tissue distribution of 4-HNE–protein adducts with their presence in the blood of patients with prostate cancer [90]. Therefore, the detection and identification of site-specific protein modification with 4-HNE is necessary to better understand the biological relevance of the 4-HNE adductome and to uncover the underlying mechanisms mediated by 4-HNE. Thus, 4-HNE–His adducts were detected as the major product of LPO in human LDL, whereas other 4-HNE-derived Michael adducts, including 4-HNE–Lys and 4-HNE–Cys adducts and 2-alkenal-derived Michael adducts were barely detected [91]. In the same work, the authors described 4-HNE–His to be the major modification of proteins exposed to 4-HNE in vitro, whereas 4-HNE–His adducts were identified as ligands for LOX-1 (lectin-like oxidized low-density lipoprotein receptor-1), which is an endothelial scavenger receptor for the uptake of oxidized LDL important in the pathogenesis of atherosclerosis [92].

Which proteins are targets of 4-HNE, in addition to those already mentioned above, appears to depend on the intensity of 4-HNE exposure and may be cell-type specific, reflecting pathophysiological conditions. For example, proteomic analysis of normal human plasma and plasma from patients with psoriasis revealed that psoriatic patients had more 4-HNE-modified proteins than controls, which were predominantly classified as signaling molecules, in contrast to healthy controls in which predominantly structural proteins were modified by 4-HNE [68]. Moreover, THP-1 cells treated with 100 µM 4-HNE, a concentration that induces cell apoptosis and necrosis, showed preferential modification of proteins involved in cytoskeletal organization/regulation (whose modulation of which may stimulate apoptosis), stress response, and glycolysis. Most modifications occurred at Cys residues and to a lesser extent at His residues. Site-specific modifications identified include: tubulin α-1B chain (Cys295 and Cys347), α-actinin-4 (Cys351 and Cys499), β-actin (His-40), vimentin (Cys328), D-3-phosphoglycerate dehydrogenase (Cys369), and aldolase A (His246) [93]. However, intracellular glutathione concentration should not be neglected when assessing changes in the 4-HNE–protein adductome [94].

Although mass spectrometry today is utilized to identify proteins sensitive to 4-HNE, the biological significance of 4-HNE adduction for many proteins is still unclear. Some effects of 4-HNE adduction to proteins are listed in Table 3.

Detailed analysis of the 4-HNE adductome will reveal amino acid residues that are susceptible to 4-HNE. Studies have shown that 4-HNE can affect enzyme function either by direct modification of the catalytic site or by adduction to residues outside the catalytic site that affects protein conformation and substrate accessibility to the catalytic site. Catalytic, transferase, and hydrolase activities are among the enzymatic activities affected by 4-HNE protein modifications. The specific residues of proteins involved in lipid binding (FABP) are particularly sensitive to impairment of function by 4-HNE and consequently may lead to impaired metabolic homeostasis. As summarized in Table 3, specific residues of proteins with antioxidant activity, such are glutamate–cysteine ligase, glutathione S-transferases, and peroxiredoxin, are impaired in function after modification with 4-HNE.

## 6. 4-HNE–Protein Adducts Impacting Cellular Homeostasis

4-HNE affects various cellular processes in a concentration-dependent manner, from proliferation and differentiation to autophagy and cell death such as apoptosis or ferroptosis [27,118,119]. Therefore, it is not surprising that 4-HNE not only plays an important role in carcinogenesis but also in the defense of normal cells against cancer [120,121,122,123,124]. Thus, it seems that the generation of 4-HNE and the way it acts are not merely accidental but rather targeted. Although exogenous addition of 4-HNE has revealed a myriad of proteins that can form adducts with 4-HNE [51,63,73], knowledge about the preferred target proteins, especially in the physiological range of 4-HNE, is still limited. Moreover, their involvement in diverse signaling pathways known to be affected by them, such as NRF2/KEAP1 (nuclear factor erythroid 2-like 2/kelch-like ECH-associated protein 1), MAPKs (mitogen-activated protein kinases), PI3K/AKT (phosphoinositide-3-kinase/protein kinase B), and NFκB (nuclear factor-κB) needs full elucidation [125,126,127,128]. To mediate a signaling pathway, 4-HNE needs not only to modify specific target but its concentration should also be controlled, keeping it in a range that will elicit stimuli-induced cellular output. The glutathione-S transferases family of enzymes plays an important role in the detoxification of 4-HNE. There are several isoforms of GST, each with different substrate specificities and cellular functions. The GST A4-4 isoform has been shown to be the most important for detoxification of 4-HNE and is characterized by its low susceptibility to 4-HNE adduction [129]. Moreover, 4-HNE itself induces the expression of GST A4-4 by activating a c-JUN/NRF2 complex [130]. This suggests that cells have evolved one or more mechanisms to control 4-HNE levels and maintain them in a range that facilitates its signaling while minimizing its toxic effects.

The formation of 4-HNE–protein adducts may have a significant impact on cellular adaptive response and cellular homeostasis. Two key signaling pathways that have been linked to 4-HNE–protein adducts are the NRF2/KEAP1 pathway and the ferroptosis pathway.

### 6.1. HNE–KEAP1 Adducts and Adaptive Response

As a response to oxidative stress, cells activate protective mechanisms. The primary one is the NRF2 signaling pathway. Although the regulation of the transcription factor NRF2 is complex, the main regulator of its activity is KEAP1, which is rich in cysteines that are sensitive to electrophiles such as 4-HNE [131,132].

**Figure 3:**
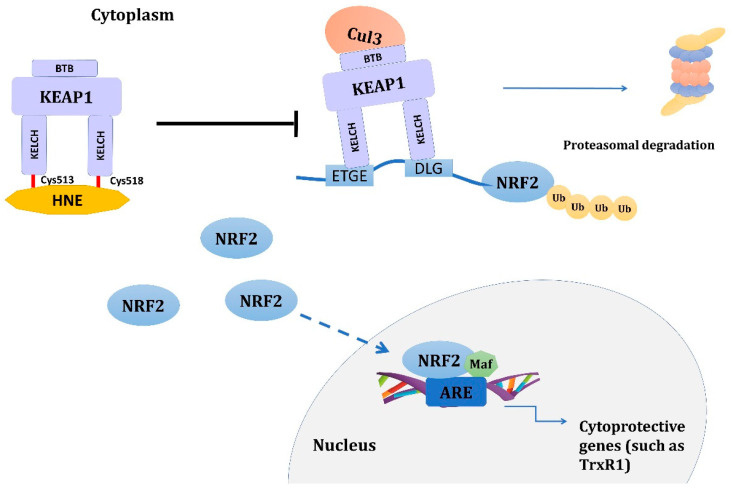
Activation of NRF2 signaling pathway by 4-HNE. 4-HNE adduction of KEAP1 at Cys513 and Cys518 disrupts the binding of NRF2 to KEAP1 and subsequent proteasomal degradation of NRF2. Instead, accumulated cytoplasmatic NRF2 translocates to the nucleus, initiating the expression of more than 200 genes containing the ARE in their promoter, such as TrxR1. Other cysteine residues in KEAP1, such as Cys151, Cys288, Cys226, and Cys368, have also been suggested to be involved in the activation of the NRF2 signaling pathway. Abbreviations: 4-HNE: 4-hydroxynonenal, ARE: antioxidant response element, BTB: Broad complex, Tramtrack, and Bric-a-Brac domain of KEAP1, Cul3: Cullin 3 E3 ubiquitin ligase, Cys: cysteine, DLG: motif of the Neh2 domain of the NRF2 responsible for direct interaction with KEAP1, ETGE: motif of the Neh2 domain of the NRF2 responsible for direct interaction with KEAP1, KEAP1: kelch-like ECH-associated protein 1, KELCH: Kelch domain of KEAP1, Maf: small Maf proteins, NRF2: nuclear factor erythroid 2-like 2, TrxR1: thioredoxin reductase 1, Ub: ubiquitin.

Chen and colleagues [133] showed that pretreatment of PC12 cells with 4-HNE protected the cells from impending oxidative stress. This cytoprotective effect was attributed to the induction of thioredoxin reductase 1 (TrxR1) through transcriptional activation of NRF2. The likely mechanism involves 4-HNE adduction of KEAP1 [134], which disrupts the NRF2 binding to KEAP1 and subsequent proteasomal degradation of NRF2. Instead, the accumulated cytoplasmic NRF2 translocates to the nucleus initiating the expression of more than 200 genes containing the antioxidant response element (ARE) in their promoters [135]; TrxR1 is one of them [136]. In fact, Gao et al. linked a deficiency of solute carrier family 27 member 5 (SLC27A5/FATP5), which leads to an increase in PUFA, to accumulation of HNE adducts and activation of the NRF2/TrxR1 axis to treatment resistance in hepatocellular carcinoma. The underlying mechanism involves the modification of Cys513 and Cys518 in the Kelch domain of KEAP1 by 4-HNE [137]. The modifications of Cys513 and Cys518, among others, are linked with the sulforaphane’s (a known activator of NRF2 signaling) mode of action [138] (Figure 3). However, other modifications of KEAP1 are also suggested to be involved in 4-HNE-induced activation of the NRF2/KEAP1 signaling pathway, such as Cys151, Cys288, Cys226, and Cys368 [139,140].

### 6.2. 4-HNE–Protein Adducts in Ferroptosis

Ferroptosis is a type of programmed cell death regulated by iron-mediated lipid peroxidation. Although it has some similarities, it differs both morphologically and biochemically from other known types of cell death. The main morphological distinction observed in ferroptotic cells is the disruption of mitochondrial structure. Biochemically, ferroptosis is characterized by insufficient glutathione peroxidase 4 (GPX4) activity and GSH depletion leading to peroxidation of the cellular membrane phospholipids in an iron-rich milieu [119]. Ferroptosis is not only associated with the pathophysiology of various diseases but is also considered a natural barrier to cancer development, although the full extent of its role in cellular homeostasis is not yet clear [141].

4-HNE plays an important role in sensitizing cells to ferroptosis. Chen et al. have shown that 4-HNE accumulates in cells through inhibition of aldehyde dehydrogenase 1B1 (ALDH1B1) by eukaryotic initiation factor 4E (EIF4E). This accumulation of 4-HNE activates NADPH oxidase 1 (NOX1), which fosters ferroptosis [142]. Further studies revealed eight endogenous sites of modification by 4-HNE, 11 sites by acrolein, and 5 sites by 4-ONE upon the induction of ferroptosis in HT1080 cells. Only the modifications of Cys210 in voltage-dependent anion-selective channel protein 2 (VDAC2) and Cys1101 of reticulon-4 (RTN4) were common to 4-HNE and 4-ONE, indicating fairly high selectivity and specificity. Other 4-HNE–protein adducts included Cys103 of VDAC2, Cys328 of vimentin (VIME), Cys477 of ribophorin 1 (RPN1), Cys71 of proline, glutamate and leucine rich protein 1 (PELP1), Cys58 of the mitochondrial contact site and cristae organizing system subunit 10 (MIC10), and Cys108 of Nipsnap homolog 2 (NIPS2). The high sensitivity of the Cys210 of VDAC2 to 4-HNE has been proposed to trigger ferroptosis [143], likely leading to mitochondrial dysfunction as observed with erastin. Interestingly, 4-HNE–protein adducts are also increased in erastin-induced ferroptosis, in which its binding to VDAC2 leads to altered mitochondrial membrane permeability, a decrease in NADH oxidation, accumulation of ROS, lipid peroxidation, and cell death [144].

However, 4-HNE not only induces ferroptosis but can also trigger resistance to ferroptosis. It has been shown that 4-HNE activates the p38/MAPK signaling pathway, leading to phosphorylation and activation of the stress-responsive transcription factor heat shock factor 1 (HSF1). This leads to an increase in the expression of prominin2, which confers resistance to ferroptosis. It was also noted that other lipid peroxidation products such as MDA, 4-ONE, and 4-HHE did not have this effect, suggesting that 4-HNE specifically regulates the expression of prominin2, although the contribution of other LPO products cannot be completely excluded [145]. Although highly speculative, the mechanism might include 4-HNE adduction of Src on Cys 248, leading to consequent activation of p38 [116] or promoting nuclear translocation of HSF1. Adduction of Cys267 HSP70 [107] and/or Cys572 or several His HSP90 [76,108] with 4-HNE confers their inhibitory effect of HSF1 [146,147].

These examples highlight the important role that 4-HNE–protein adducts play in regulating the cellular adaptive response and cellular homeostasis. Further research is needed to fully understand the mechanisms by which 4-HNE–protein adducts influence these signaling pathways and how these effects contribute to cellular stress, disease, and therapy resistance.

## 7. Conclusions

The LPO-derived aldehyde 4-HNE is undoubtedly a bioactive molecule that can either directly or indirectly modulate various cellular processes in both physiology and pathology. The most common methods used for the detection of 4-HNE–protein adducts are immunochemical methods, where the detection and selection of proteins modified by 4-HNE relies on the specificity of the 4-HNE antibodies used and subsequent identification by mass spectrometry. With the development of technologies, new methods have emerged for direct analysis of site-specific 4-HNE protein modifications with minimal sample manipulation. Today, a number of proteins have been found to be susceptible to adduction with 4-HNE; however, the biological relevance of such modifications is frequently unclear. Depending on the protein amino acid site adducted with 4-HNE, the protein structure and function may be altered. The examples of the importance of 4-HNE–protein adducts in regulating cellular adaptive responses and cellular homeostasis highlight the need for a deeper understanding of the mechanisms by which 4-HNE–protein adducts impact these signaling pathways. The effects of 4-HNE–protein adducts on cellular stress, disease, and therapy resistance are still not fully understood. Therefore, to understand the biological relevance of the 4-HNE adductome, the identification of modified proteins should be accompanied by the studies exploring the involvement of such modifications on targeted protein structure and function.

## Figures and Tables

**Table 1:** Commercially available monoclonal antibodies.

Clone	Immunogen	Applications	Description	Specificity
**HNEJ-2**	4-HNE-modified KLH	IHC, WB, ELISA	mouse IgG1 kappa	Recognizes 4-HNE; Negligible reactivity with proteins that were treated with other aldehydes, such as 2-nonenal, 2-hexenal, 1-hexanal, 4-hydroxyhexenal, formaldehyde, or glutaraldehyde.
**12F7**	4-HNE-modified KLH	WB, IHC, ICC/IF, ELISA	mouse IgG1	Specific for 4-HNE-modified proteins. Does not detect free 4-HNE. Does not react with 4-hydroxyhexenal, acrolein, crotonaldehyde, hexanoyl lys, MDA, or methylglyoxal-modified proteins
**198960**	4-HNE-modified KLH	WB, IHC, Simple Western	mouse IgG2b	Detects 4-hydroxynonenal adducts of histidine residues. No cross-reactivity with nitrotyrosine, formaldehyde, glutaraldehyde, or 4-hydroxy hexenaldehyde adducts is observed.
**9H132**	4-HNE-modified KLH	WB	mouse IgG2b	Recognizes 4-HNE adducts of histidine residues. It does not cross-react with nitrotyrosine, formaldehyde, glutaraldehyde, or 4-hydroxy hexenaldehyde adducts.
**IOFK-3**	4-HNE-modified KLH	WB, IHC	mouse	This antibody shows almost negligible reactivity with proteins that were treated with other aldehydes such as: 2-nonenal, 2-hexenal, 1-hexenal, 4-hydroxy-2-hexenal, formaldehyde, or glutaraldehyde.

Abbreviations: KLH—keyhole limpet hemocyanin, 4-HNE—4-hydroxynonenal, IHC—immunohistochemistry, ICC—immunocytochemistry, IF—immunofluorescence, WB—Western blot; MDA—malondialdehyde.

**Table 2:** Analysis of 4-HNE–protein adducts in complex samples by mass spectrometry.

Sample Type	Sample Preparation	Method (Mass Analyzer)	Adducts Searched	Ref.
Adipocytes	Nuclear proteins, immunoprecipitation of carbonylated proteins, trypsin digestion	NanoLC-ESI-CID-MS/MS (Orbitrap)	4-HNE–Cys, 4-HNE–His, 4-HNE–Lys	[58]
Chondrocytes	Whole cell lysates, nuclear and cytosolic protein extracts, trypsin or pepsin digestion followed by SPE	UHPLC-QTOF-MS/MS, TOF-MS	4-HNE–Cys, 4-HNE–His, 4-HNE–Lys	[61]
Heart (left ventricle) samples	Whole tissue lysate, SDS-PAGE, trypsin digestion	NanoLC-ESI-HCD-MS/MS (Orbitrap and Iontrap), neutral ion loss (46, 52, 69, 78 *m*/*z*) triggered ETD-Iontrap MS/MS	4-HNE–Cys, 4-HNE–His, 4-HNE–Lys, 4-HNE–Arg	[62]
HEK293T cells	Cell lysate, protein labeling, click chemistry, protein digestion	LC-HCD-MS/MS (Orbitrap)	Modification of Cys, His, and Lys	[63]
Keratinocytes and lymphocytes	Whole cell lysates, SDS-PAGE, trypsin digestion	LC-ESI-HCD-MS/MS (Quadrupole-Orbitrap)	4-HNE–Cys, 4-HNE–His, 4-HNE–Lys	[64]
MCF-7 cells	Whole cell lysate, trypsin digestion, fluorous derivatization, fluorous SPE	UPLC-ESI-HCD-MS/MS (Quadrupole-Orbitrap)	4-HNE–Cys, 4-HNE–His, 4-HNE–Lys, 4-HNE–Arg	[65]
MDA-MB-231	Cell lysate, protein labeling, click chemistry, protein digestion	LC-MS/MS	4-HNE–Cys	[66]
Mice white adipose depots; Drosophila Melanogaster Flies	Histone purification, immunoprecipitation of 4-HNE-modified proteins, SDS-PAGE, trypsin digestion	NanoLC-ESI-CID-MS/MS (Orbitrap and Iontrap)	4-HNE–Cys, 4-HNE–His, 4-HNE–Lys	[67]
Plasma samples	Albumin removal, SDS-PAGE, trypsin digestion	LC-ESI-HCD-MS/MS (Quadrupole-Orbitrap)	4-HNE–Cys, 4-HNE–His, 4-HNE–Lys	[68]
Rat, mouse, and human liver microsomes	Trypsin digestion followed by SPE clean-up, fractionation by HPLC-DAD	UHPLC-ESI-MS/MS (Quadrupole-TOF)	4-HNE–Cys, 4-HNE–His, 4-HNE–Lys	[69]
Rat retina	S2-DE, WB, band excision, digestion with digested with 0.005% tosylsulfonyl phenylalanyl chloromethyl ketone-treated trypsin	MALDI-TOF/TOF	n.s.	[70]
RKO human colorectal cancer cells	Lysate of transfected cells and purification of CDK2–His, SDS-PAGE, selection of gel bands corresponding to CDK2–His, trypsin digestion	HPLC-ESI-MS/MS (Orbitrap and linear trap quadrupole)	4-HNE–Cys, 4-HNE–His, 4-HNE–Lys, 4-HNE–Arg	[71]
Serum samples; Skeletal muscle cells; mouse pancreatic islets	Immunoprecipitation of 4-HNE-modified proteins, trypsin digestion	HPLC-ESI-TOF MS/MS	n.s.	[72]

Abbreviations: 2-DE—two-dimensional gel electrophoresis; DAD—diode-array detector; SPE—solid phase extraction; TOF—time of flight.

**Table 3:** Biological relevance of HNE-protein adducts detected by mass spectrometry.

Protein	Site Modified (Detected)	The Effect of 4-HNE Adduction	Ref.
Adipocyte fatty-acid-binding protein (A-FABP)	Cys117	Decreases affinity for fatty acids.	[95]
Akt2	His196, His267, Cys311	Inhibits insulin-dependent Akt signaling by restricting substrate binding to Akt2.	[96]
Alpha-synuclein	His50 **	Induces α-synuclein oligomerization and stabilizes it against dissociation to monomers.	[48]
Brain cytosolic isoform of creatine kinase (CK-BB)	His7, His26, His29, His66 *, Lys86, His97, Lys101, Cys141, Cys145, His191 *, His234, Cys254, His276, Cys283 *, His296 *, His305	Dose-dependent reduction in enzyme activity; Cys283 is readily modified by 4-HNE even at low concentrations.	[97]
Cathepsin B	Cys29 (A chain), His150 (B chain)	Inactivation of the enzyme and loss of protease activity.	[98]
Cyclin-dependent kinase 2 (CDK2)	His60, His71 *, Lys129, His161 *, His268, His283, His295	Decreases kinase activity and leads to cell cycle arrest.	[71]
Cytochrome c	Arg38, His33, Lys87	Decreases protein pI and structure and could affect mitochondrial function.	[99]
Cytochrome c oxidase subunit VIII	His36	Inactivation of the enzyme	[100]
Epithelial fatty acid-binding protein (E-FABP)	Lys115, Cys120 *, Cys127	Stabilizes the E-FABP structure against chemical denaturation by guanidine hydrochloride.	[101]
Extracellular signal regulated kinase (Erk-1/2)	His178	Modification of inactive cytosolic monomers results in inhibition of Erk-1/2 phosphorylation and activity.	[102]
Glucose-6-phosphate dehydrogenase (G6PD)	Lys (n.s.)	Inactivation of the enzyme	[103]
Glutamate–cysteine ligase (GCL)	GCLC subunit (Cys553) GCLM subunit (Cys35)	Increases the activity of the cellular enzyme. Increased activity of monomeric GCLC. Modified GCLM may affect formation of GCL holoenzyme.	[104]
Glutathione S-transferases (GST) isoforms: alpha (GSTA), mu (GSTM) and pi (GSTP)	n.s.	Decreases the catalytic activity of GST with 1-chloro-2,4-dinitrobenzene as substrate. The strongest effect was recorded for GSTP, where extensive modification with 4-HNE was accompanied by an almost complete loss of enzyme activity.	[105]
Glyceraldehyde-3-phosphate dehydrogenase (GAPDH)	His164, Cys244, Cys281, His327, Lys331	The modifications detected indicate that the inactivation of GAPDH by 4-HNE is not due to modification of the active site but is likely due to impairment of the protein structure.	[106]
Heat shock protein 72-kDa (Hsp72)	Cys267	Reduced Hsp72-mediated protein refolding efficiency and decreased affinity for ATP.	[107]
Heat shock protein 90-kDa (Hsp90)	Cys572	Inhibition of Hsp90-mediated chaperone activity	[108]
Human serum albumin (HSA)	Cys34, His67, His146, Lys 195, Lys 199, His242, His288, His510, Lys525	Cys34 is the residue most susceptible to 4-HNE adduction, which could impair HSA function.	[109]
Liver fatty acid-binding protein (L-FABP), unbound apo and lipid-bound holo	Apo L-FABP (Lys57, Cys69) Holo L-FABP (Lys6, Lys31, His43, Lys46, Lys57, Cys69)	Binding capacity for natural ligands is reduced.	[110]
Mitochondrial aldehyde dehydrogenase (ALDH2)	Cys302 *	Irreversible inhibition of enzyme activity at very high concentrations of the aldehyde when the active site is modified (500 μM), but reversible at lower concentrations (50 μM).	[111]
Peroxiredoxin 6 (PRX6)	Cys91	Induces a distortion in the tertiary protein structure and alters the conformation of the active site.	[112]
Phosphatase and tensin homolog deleted on chromosome 10 (PTEN)	Cys71 *, Cys136, Lys147, Lys223, Cys250, Lys254, Lys313, Lys327 *, Lys344	Inhibition of enzyme activity	[113]
Pyruvate kinase M2 isoform (PKM2)	Cys49, His274, Cys424 *, His439 *, Lys256	Inhibits kinase activity. Cys424 is involved in protein-protein interactions, whereas it is His439 in fructose 1,6-bis-phosphate binding.	[114]
Sirtuin 3 (SIRT3)	Cys280	Allosteric inhibition of SIRT3 activity.	[115]
Src Tyrosine Kinase (Src)	His236, Cys241, Cys248 *	Activation of Src by adduct formation at Cys248, which induces autophosphorylation of Tyr416.	[116]
Tubulin	α-Tubulin (Cys347, Cys376) β-Tubulin (Cys303)	Impairment of tubulin polymerization	[117]
ZAK kinase	Cys22 *	Inhibition of enzyme activity, resulting in a negative feedback mechanism that may reduce activation of the JNK pathway.	[66]

* Residues with catalytic activity and function. ** His50 is predominant site, although a number of 4-HNE–Lys residues were detected.

## Data Availability

Not applicable.

## References

[1] Sies H., Sies H. (1985). Oxidative Stress: Introductory Remarks. Oxidative Stress.

[2] Pizzino G., Irrera N., Cucinotta M., Pallio G., Mannino F., Arcoraci V., Squadrito F., Altavilla D., Bitto A. (2017). Oxidative Stress: Harms and Benefits for Human Health. Oxid. Med. Cell. Longev..

[3] Ramani S., Pathak A., Dalal V., Paul A., Biswas S. (2020). Oxidative Stress in Autoimmune Diseases: An Under Dealt Malice. Curr. Protein Pept. Sci..

[4] Milkovic L., Cipak Gasparovic A., Cindric M., Mouthuy P.A., Zarkovic N. (2019). Short Overview of ROS as Cell Function Regulators and Their Implications in Therapy Concepts. Cells.

[5] Sies H. (2018). On the history of oxidative stress: Concept and some aspects of current development. Curr. Opin. Toxicol..

[6] Sies H., Belousov V.V., Chandel N.S., Davies M.J., Jones D.P., Mann G.E., Murphy M.P., Yamamoto M., Winterbourn C. (2022). Defining roles of specific reactive oxygen species (ROS) in cell biology and physiology. Nat. Rev. Mol. Cell Biol..

[7] Jaganjac M., Cindrić M., Jakovčević A., Žarković K., Žarković N. (2021). Lipid peroxidation in brain tumors. Neurochem. Int..

[8] Esterbauer H., Schaur R.J., Zollner H. (1991). Chemistry and biochemistry of 4-hydroxynonenal, malonaldehyde and related aldehydes. Free Radic. Biol. Med..

[9] Ayala A., Muñoz M.F., Argüelles S. (2014). Lipid peroxidation: Production, metabolism, and signaling mechanisms of malondialdehyde and 4-hydroxy-2-nonenal. Oxid. Med. Cell. Longev..

[10] Jin J., Zheng Y., Brash A.R. (2013). Demonstration of HNE-related aldehyde formation via lipoxygenase-catalyzed synthesis of a bis-allylic dihydroperoxide intermediate. Chem. Res. Toxicol..

[11] Schneider C., Tallman K.A., Porter N.A., Brash A.R. (2001). Two distinct pathways of formation of 4-hydroxynonenal. Mechanisms of nonenzymatic transformation of the 9- and 13-hydroperoxides of linoleic acid to 4-hydroxyalkenals. J. Biol. Chem..

[12] Bilska-Wilkosz A., Iciek M., Górny M. (2022). Chemistry and Biochemistry Aspects of the 4-Hydroxy-2,3-trans-nonenal. Biomolecules.

[13] Liu W., Porter N.A., Schneider C., Brash A.R., Yin H. (2011). Formation of 4-hydroxynonenal from cardiolipin oxidation: Intramolecular peroxyl radical addition and decomposition. Free Radic. Biol. Med..

[14] Schaur R.J., Siems W., Bresgen N., Eckl P.M. (2015). 4-Hydroxy-nonenal-A Bioactive Lipid Peroxidation Product. Biomolecules.

[15] Siems W., Grune T. (2003). Intracellular metabolism of 4-hydroxynonenal. Mol. Aspects Med..

[16] Bruenner B.A., Jones A.D., German J.B. (1995). Direct characterization of protein adducts of the lipid peroxidation product 4-hydroxy-2-nonenal using electrospray mass spectrometry. Chem. Res. Toxicol..

[17] Doorn J.A., Petersen D.R. (2002). Covalent modification of amino acid nucleophiles by the lipid peroxidation products 4-hydroxy-2-nonenal and 4-oxo-2-nonenal. Chem. Res. Toxicol..

[18] Lesgards J.-F., Frayne I.R., Comte B., Busseuil D., Rhéaume E., Tardif J.-C., Rosiers C. (2009). Des Differential distribution of 4-hydroxynonenal adducts to sulfur and nitrogen residues in blood proteins as revealed using Raney nickel and gas chromatography-mass spectrometry. Free Radic. Biol. Med..

[19] LoPachin R.M., Geohagen B.C., Gavin T. (2009). Synaptosomal Toxicity and Nucleophilic Targets of 4-Hydroxy-2-Nonenal. Toxicol. Sci..

[20] Sauerland M., Mertes R., Morozzi C., Eggler A.L., Gamon L.F., Davies M.J. (2021). Kinetic assessment of Michael addition reactions of alpha, beta-unsaturated carbonyl compounds to amino acid and protein thiols. Free Radic. Biol. Med..

[21] Aslebagh R., Pfeffer B.A., Fliesler S.J., Darie C.C. (2016). Mass spectrometry-based proteomics of oxidative stress: Identification of 4-hydroxy-2-nonenal (HNE) adducts of amino acids using lysozyme and bovine serum albumin as model proteins. Electrophoresis.

[22] Annangudi S.P., Deng Y., Gu X., Zhang W., Crabb J.W., Salomon R.G. (2008). Low-density lipoprotein has an enormous capacity to bind (E)-4-hydroxynon-2-enal (HNE): Detection and characterization of lysyl and histidyl adducts containing multiple molecules of HNE. Chem. Res. Toxicol..

[23] Wakita C., Maeshima T., Yamazaki A., Shibata T., Ito S., Akagawa M., Ojika M., Yodoi J., Uchida K. (2009). Stereochemical configuration of 4-hydroxy-2-nonenal-cysteine adducts and their stereoselective formation in a redox-regulated protein. J. Biol. Chem..

[24] Niki E. (2016). Oxidative stress and antioxidants: Distress or eustress?. Arch. Biochem. Biophys..

[25] Castro J.P., Jung T., Grune T., Siems W. (2017). 4-Hydroxynonenal (HNE) modified proteins in metabolic diseases. Free Radic. Biol. Med..

[26] Carbone D.L., Doorn J.A., Kiebler Z., Petersen D.R. (2005). Cysteine modification by lipid peroxidation products inhibits protein disulfide isomerase. Chem. Res. Toxicol..

[27] Milkovic L., Cipak Gasparovic A., Zarkovic N. (2015). Overview on major lipid peroxidation bioactive factor 4-hydroxynonenal as pluripotent growth-regulating factor. Free Radic. Res..

[28] Jaganjac M., Milkovic L., Gegotek A., Cindric M., Zarkovic K., Skrzydlewska E., Zarkovic N. (2020). The relevance of pathophysiological alterations in redox signaling of 4-hydroxynonenal for pharmacological therapies of major stress-associated diseases. Free Radic. Biol. Med..

[29] Zhang H., Forman H.J. (2017). Signaling by 4-hydroxy-2-nonenal: Exposure protocols, target selectivity and degradation. Arch. Biochem. Biophys..

[30] Riahi Y., Cohen G., Shamni O., Sasson S. (2010). Signaling and cytotoxic functions of 4-hydroxyalkenals. Am. J. Physiol. Endocrinol. Metab..

[31] Palinski W., Ylä-Herttuala S., Rosenfeld M.E., Butler S.W., Socher S.A., Parthasarathy S., Curtiss L.K., Witztum J.L. (1990). Antisera and monoclonal antibodies specific for epitopes generated during oxidative modification of low density lipoprotein. Arterioscler. Off. J. Am. Heart Assoc. Inc..

[32] Jürgens G., Ashy A., Esterbauer H. (1990). Detection of new epitopes formed upon oxidation of low-density lipoprotein, lipoprotein (a) and very-low-density lipoprotein. Use of an antiserum against 4-hydroxynonenal-modified low-density lipoprotein. Biochem. J..

[33] Chen Q., Esterbauer H., Jürgens G. (1992). Studies on epitopes on low-density lipoprotein modified by 4-hydroxynonenal. Biochemical characterization and determination. Biochem. J..

[34] Uchida K., Toyokuni S., Nishikawa K., Kawakishi S., Oda H., Hiai H., Stadtman E.R. (1994). Michael Addition-Type 4-Hydroxy-2-nonenal Adducts in Modified Low-Density Lipoproteins: Markers for Atherosclerosis. Biochemistry.

[35] Uchida K., Szweda L.I., Chae H.Z., Stadtman E.R. (1993). Immunochemical detection of 4-hydroxynonenal protein adducts in oxidized hepatocytes. Proc. Natl. Acad. Sci. USA.

[36] Toyokuni S., Miyake N., Hiai H., Hagiwara M., Kawakishi S., Osawa T., Uchida K. (1995). The monoclonal antibody specific for the 4-hydroxy-2-nonenal histidine adduct. FEBS Lett..

[37] Waeg G., Dimsity G., Esterbauer H. (1996). Monoclonal antibodies for detection of 4-hydroxynonenal modified proteins. Free Radic. Res..

[38] Uchida K., Stadtman E.R. (1992). Modification of histidine residues in proteins by reaction with 4-hydroxynonenal. Proc. Natl. Acad. Sci. USA.

[39] Weber D., Milkovic L., Bennett S.J., Griffiths H.R., Zarkovic N., Grune T. (2013). Measurement of HNE-protein adducts in human plasma and serum by ELISA-Comparison of two primary antibodies. Redox Biol..

[40] Hartley D.P., Kroll D.J., Petersen D.R. (1997). Prooxidant-initiated lipid peroxidation in isolated rat hepatocytes: Detection of 4-hydroxynonenal- and malondialdehyde-protein adducts. Chem. Res. Toxicol..

[41] Campos-Pinto I., Méndez L., Schouten J., Wilkins J., Fedorova M., Pitt A.R., Davis P., Spickett C.M. (2019). Epitope mapping and characterization of 4-hydroxy-2-nonenal modified-human serum albumin using two different polyclonal antibodies. Free Radic. Biol. Med..

[42] Zarkovic K., Jakovcevic A., Zarkovic N. (2017). Contribution of the HNE-immunohistochemistry to modern pathological concepts of major human diseases. Free Radic. Biol. Med..

[43] Živković M., Žarković K., Škrinjar L., Waeg G., Poljak-Blaži M., Šunjić S.B., Schaur R.J., Žarković N. (2005). A new method for detection of HNE-histidine conjugates in rat inflammatory cells. Croat. Chem. Acta.

[44] Borovic S., Rabuzin F., Waeg G., Zarkovic N. (2006). Enzyme-linked immunosorbent assay for 4-hydroxynonenal-histidine conjugates. Free Radic. Res..

[45] Zhao Y., Miriyala S., Miao L., Mitov M., Schnell D., Dhar S.K., Cai J., Klein J.B., Sultana R., Butterfield D.A. (2014). Redox proteomic identification of HNE-bound mitochondrial proteins in cardiac tissues reveals a systemic effect on energy metabolism after doxorubicin treatment. Free Radic. Biol. Med..

[46] Spickett C.M., Wiswedel I., Siems W., Zarkovic K., Zarkovic N. (2010). Advances in methods for the determination of biologically relevant lipid peroxidation products. Free Radic. Res..

[47] Breusing N., Grune T., Andrisic L., Atalay M., Bartosz G., Biasi F., Borovic S., Bravo L., Casals I., Casillas R. (2010). An inter-laboratory validation of methods of lipid peroxidation measurement in UVA-treated human plasma samples. Free Radic. Res..

[48] Andersen C., Grønnemose A.L., Pedersen J.N., Nowak J.S., Christiansen G., Nielsen J., Mulder F.A.A., Otzen D.E., Jørgensen T.J.D. (2021). Lipid Peroxidation Products HNE and ONE Promote and Stabilize Alpha-Synuclein Oligomers by Chemical Modifications. Biochemistry.

[49] Williams M.V., Wishnok J.S., Tannenbaum S.R. (2007). Covalent adducts arising from the decomposition products of lipid hydroperoxides in the presence of cytochrome c. Chem. Res. Toxicol..

[50] Yang J., Tallman K.A., Porter N.A., Liebler D.C. (2015). Quantitative chemoproteomics for site-specific analysis of protein alkylation by 4-hydroxy-2-nonenal in cells. Anal. Chem..

[51] Tzeng S.-C., Maier C.S. (2016). Label-Free Proteomics Assisted by Affinity Enrichment for Elucidating the Chemical Reactivity of the Liver Mitochondrial Proteome toward Adduction by the Lipid Electrophile 4-hydroxy-2-nonenal (HNE). Front. Chem..

[52] Arashiki N., Otsuka Y., Ito D., Yang M., Komatsu T., Sato K., Inaba M. (2010). The covalent modification of spectrin in red cell membranes by the lipid peroxidation product 4-hydroxy-2-nonenal. Biochem. Biophys. Res. Commun..

[53] Sultana R., Perluigi M., Butterfield D.A. (2013). Lipid peroxidation triggers neurodegeneration: A redox proteomics view into the Alzheimer disease brain. Free Radic. Biol. Med..

[54] Atalay S., Gęgotek A., Skrzydlewska E. (2021). Protective Effects of Cannabidiol on the Membrane Proteome of UVB-Irradiated Keratinocytes. Antioxidants.

[55] Just J., Jung T., Friis N.A., Lykkemark S., Drasbek K., Siboska G., Grune T., Kristensen P. (2015). Identification of an unstable 4-hydroxynoneal modification on the 20S proteasome subunit α7 by recombinant antibody technology. Free Radic. Biol. Med..

[56] Colzani M., Criscuolo A., Casali G., Carini M., Aldini G. (2016). A method to produce fully characterized ubiquitin covalently modified by 4-hydroxy-nonenal, glyoxal, methylglyoxal, and malondialdehyde. Free Radic. Res..

[57] Pillon N.J., Vella R.E., Souleere L., Becchi M., Lagarde M., Soulage C.O. (2011). Structural and functional changes in human insulin induced by the lipid peroxidation byproducts 4-hydroxy-2-nonenal and 4-hydroxy-2-hexenal. Chem. Res. Toxicol..

[58] Hauck A.K., Zhou T., Hahn W., Petegrosso R., Kuang R., Chen Y., Bernlohr D.A. (2018). Obesity-induced protein carbonylation in murine adipose tissue regulates the DNA-binding domain of nuclear zinc finger proteins. J. Biol. Chem..

[59] Galligan J.J., Fritz K.S., Backos D.S., Shearn C.T., Smathers R.L., Jiang H., MacLean K.N., Reigan P.R., Petersen D.R. (2014). Oxidative stress-mediated aldehyde adduction of GRP78 in a mouse model of alcoholic liver disease: Functional independence of ATPase activity and chaperone function. Free Radic. Biol. Med..

[60] Jaganjac M., Milkovic L., Zarkovic N., Zarkovic K. (2022). Oxidative stress and regeneration. Free Radic. Biol. Med..

[61] Geib T., Iacob C., Jribi R., Fernandes J., Benderdour M., Sleno L. (2021). Identification of 4-hydroxynonenal-modified proteins in human osteoarthritic chondrocytes. J. Proteomics.

[62] Al-Menhali A.S., Anderson C., Gourine A.V., Abramov A.Y., D’Souza A., Jaganjac M. (2021). Proteomic Analysis of Cardiac Adaptation to Exercise by High Resolution Mass Spectrometry. Front. Mol. Biosci..

[63] Chen Y., Cong Y., Quan B., Lan T., Chu X., Ye Z., Hou X., Wang C. (2017). Chemoproteomic profiling of targets of lipid-derived electrophiles by bioorthogonal aminooxy probe. Redox Biol..

[64] Gęgotek A., Domingues P., Wroński A., Ambrożewicz E., Skrzydlewska E. (2019). The Proteomic Profile of Keratinocytes and Lymphocytes in Psoriatic Patients. Proteomics. Clin. Appl..

[65] Yuan W., Zhang Y., Xiong Y., Tao T., Wang Y., Yao J., Zhang L., Yan G., Bao H., Lu H. (2017). Highly Selective and Large Scale Mass Spectrometric Analysis of 4-Hydroxynonenal Modification via Fluorous Derivatization and Fluorous Solid-Phase Extraction. Anal. Chem..

[66] Wang C., Weerapana E., Blewett M.M., Cravatt B.F. (2014). A chemoproteomic platform to quantitatively map targets of lipid-derived electrophiles. Nat. Methods.

[67] Hauck A.K., Zhou T., Upadhyay A., Sun Y., O’connor M.B., Chen Y., Bernlohr D.A. (2020). Histone carbonylation is a redox-regulated epigenomic mark that accumulates with obesity and aging. Antioxidants.

[68] Gęgotek A., Domingues P., Wroński A., Wójcik P., Skrzydlewska E. (2018). Proteomic plasma profile of psoriatic patients. J. Pharm. Biomed. Anal..

[69] Golizeh M., Geib T., Sleno L. (2016). Identification of 4-hydroxynonenal protein targets in rat, mouse and human liver microsomes by two-dimensional liquid chromatography/tandem mass spectrometry. Rapid Commun. Mass Spectrom..

[70] Tanito M., Haniu H., Elliott M.H., Singh A.K., Matsumoto H., Anderson R.E. (2006). Identification of 4-hydroxynonenal-modified retinal proteins induced by photooxidative stress prior to retinal degeneration. Free Radic. Biol. Med..

[71] Camarillo J.M., Rose K.L., Galligan J.J., Xu S., Marnett L.J. (2016). Covalent Modification of CDK2 by 4-Hydroxynonenal as a Mechanism of Inhibition of Cell Cycle Progression. Chem. Res. Toxicol..

[72] Lavilla C.J., Billacura M.P., Hanna K., Boocock D.J., Coveney C., Miles A.K., Foulds G.A., Murphy A., Tan A., Jackisch L. (2021). Carnosine protects stimulus-secretion coupling through prevention of protein carbonyl adduction events in cells under metabolic stress. Free Radic. Biol. Med..

[73] Codreanu S.G., Zhang B., Sobecki S.M., Billheimer D.D., Liebler D.C. (2009). Global analysis of protein damage by the lipid electrophile 4-hydroxy-2-nonenal. Mol. Cell. Proteomics.

[74] Vila A., Tallman K.A., Jacobs A.T., Liebler D.C., Porter N.A., Marnett L.J. (2008). Identification of Protein Targets of 4-Hydroxynonenal Using Click Chemistry for ex Vivo Biotinylation of Azido and Alkynyl Derivatives. Chem. Res. Toxicol..

[75] Kim H.-Y.H., Tallman K.A., Liebler D.C., Porter N.A. (2009). An azido-biotin reagent for use in the isolation of protein adducts of lipid-derived electrophiles by streptavidin catch and photorelease. Mol. Cell. Proteomics.

[76] Connor R.E., Marnett L.J., Liebler D.C. (2011). Protein-selective capture to analyze electrophile adduction of hsp90 by 4-hydroxynonenal. Chem. Res. Toxicol..

[77] Zarkovic N., Cipak A., Jaganjac M., Borovic S., Zarkovic K. (2013). Pathophysiological relevance of aldehydic protein modifications. J. Proteomics.

[78] Al-Menhali A.S., Banu S., Angelova P.R., Barcaru A., Horvatovich P., Abramov A.Y., Jaganjac M. (2020). Lipid peroxidation is involved in calcium dependent upregulation of mitochondrial metabolism in skeletal muscle. Biochim. Biophys. Acta Gen. Subj..

[79] Maniti O., François-Moutal L., Lecompte M.-F., Vial C., Lagarde M., Guichardant M., Marcillat O., Granjon T. (2015). Protein “amyloid-like” networks at the phospholipid membrane formed by 4-hydroxy-2-nonenal-modified mitochondrial creatine kinase. Mol. Membr. Biol..

[80] Larroque-Cardoso P., Mucher E., Grazide M.-H., Josse G., Schmitt A.-M., Nadal-Wolbold F., Zarkovic K., Salvayre R., Nègre-Salvayre A. (2014). 4-Hydroxynonenal impairs transforming growth factor-β1-induced elastin synthesis via epidermal growth factor receptor activation in human and murine fibroblasts. Free Radic. Biol. Med..

[81] Mihalas B.P., Bromfield E.G., Sutherland J.M., De Iuliis G.N., McLaughlin E.A., John Aitken R., Nixon B. (2018). Oxidative damage in naturally aged mouse oocytes is exacerbated by dysregulation of proteasomal activity. J. Biol. Chem..

[82] Retamal M.A., Fiori M.C., Fernandez-Olivares A., Linsambarth S., Peña F., Quintana D., Stehberg J., Altenberg G.A. (2020). 4-Hydroxynonenal induces Cx46 hemichannel inhibition through its carbonylation. Biochim. Biophys. Acta Mol. Cell Biol. Lipids.

[83] Elrayess M.A., Almuraikhy S., Kafienah W., Al-Menhali A., Al-Khelaifi F., Bashah M., Zarkovic K., Zarkovic N., Waeg G., Alsayrafi M. (2017). 4-hydroxynonenal causes impairment of human subcutaneous adipogenesis and induction of adipocyte insulin resistance. Free Radic. Biol. Med..

[84] Jaganjac M., Almuraikhy S., Al-Khelaifi F., Al-Jaber M., Bashah M., Mazloum N.A., Zarkovic K., Zarkovic N., Waeg G., Kafienah W. (2017). Combined metformin and insulin treatment reverses metabolically impaired omental adipogenesis and accumulation of 4-hydroxynonenal in obese diabetic patients. Redox Biol..

[85] Žarković N., Orehovec B., Milković L., Baršić B., Tatzber F., Wonisch W., Tarle M., Kmet M., Mataić A., Jakovčević A. (2021). Preliminary Findings on the Association of the Lipid Peroxidation Product 4-Hydroxynonenal with the Lethal Outcome of Aggressive COVID-19. Antioxidants.

[86] Zarkovic N., Jakovcevic A., Mataic A., Jaganjac M., Vukovic T., Waeg G., Zarkovic K. (2022). Post-mortem Findings of Inflammatory Cells and the Association of 4-Hydroxynonenal with Systemic Vascular and Oxidative Stress in Lethal COVID-19. Cells.

[87] Mahdi A., Collado A., Tengbom J., Jiao T., Wodaje T., Johansson N., Farnebo F., Färnert A., Yang J., Lundberg J.O. (2022). Erythrocytes Induce Vascular Dysfunction in COVID-19. JACC. Basic Transl. Sci..

[88] Casós K., Zaragozá M.C., Zarkovic N., Zarkovic K., Andrisic L., Portero-Otín M., Cacabelos D., Mitjavila M.T. (2010). A fish-oil-rich diet reduces vascular oxidative stress in apoE(-/-) mice. Free Radic. Res..

[89] Zarkovic K., Larroque-Cardoso P., Pucelle M., Salvayre R., Waeg G., Nègre-Salvayre A., Zarkovic N. (2015). Elastin aging and lipid oxidation products in human aorta. Redox Biol..

[90] Perkovic M.N., Jaganjac M., Milkovic L., Horvat T., Rojo D., Zarkovic K., Ćorić M., Hudolin T., Waeg G., Orehovec B. (2023). Relationship between 4-Hydroxynonenal (4-HNE) as Systemic Biomarker of Lipid Peroxidation and Metabolomic Profiling of Patients with Prostate Cancer. Biomolecules.

[91] Wakita C., Honda K., Shibata T., Akagawa M., Uchida K. (2011). A method for detection of 4-hydroxy-2-nonenal adducts in proteins. Free Radic. Biol. Med..

[92] Kumano-Kuramochi M., Shimozu Y., Wakita C., Ohnishi-Kameyama M., Shibata T., Matsunaga S., Takano-Ishikawa Y., Watanabe J., Goto M., Xie Q. (2012). Identification of 4-hydroxy-2-nonenal-histidine adducts that serve as ligands for human lectin-like oxidized LDL receptor-1. Biochem. J..

[93] Chavez J., Chung W.-G., Miranda C.L., Singhal M., Stevens J.F., Maier C.S. (2010). Site-Specific Protein Adducts of 4-Hydroxy-2(E)-Nonenal in Human THP-1 Monocytic Cells: Protein Carbonylation Is Diminished by Ascorbic Acid. Chem. Res. Toxicol..

[94] Codreanu S.G., Ullery J.C., Zhu J., Tallman K.A., Beavers W.N., Porter N.A., Marnett L.J., Zhang B., Liebler D.C. (2014). Alkylation damage by lipid electrophiles targets functional protein systems. Mol. Cell. Proteomics.

[95] Grimsrud P.A., Picklo M.J.S., Griffin T.J., Bernlohr D.A. (2007). Carbonylation of adipose proteins in obesity and insulin resistance: Identification of adipocyte fatty acid-binding protein as a cellular target of 4-hydroxynonenal. Mol. Cell. Proteomics.

[96] Shearn C.T., Fritz K.S., Reigan P., Petersen D.R. (2011). Modification of Akt2 by 4-hydroxynonenal inhibits insulin-dependent Akt signaling in HepG2 cells. Biochemistry.

[97] Eliuk S.M., Renfrow M.B., Shonsey E.M., Barnes S., Kim H. (2007). Active site modifications of the brain isoform of creatine kinase by 4-hydroxy-2-nonenal correlate with reduced enzyme activity: Mapping of modified sites by Fourier transform-ion cyclotron resonance mass spectrometry. Chem. Res. Toxicol..

[98] Crabb J.W., O’Neil J., Miyagi M., West K., Hoff H.F. (2002). Hydroxynonenal inactivates cathepsin B by forming Michael adducts with active site residues. Protein Sci..

[99] Isom A.L., Barnes S., Wilson L., Kirk M., Coward L., Darley-Usmar V. (2004). Modification of Cytochrome c by 4-hydroxy-2-nonenal: Evidence for histidine, lysine, and arginine-aldehyde adducts. J. Am. Soc. Mass Spectrom..

[100] Musatov A., Carroll C.A., Liu Y.-C., Henderson G.I., Weintraub S.T., Robinson N.C. (2002). Identification of bovine heart cytochrome c oxidase subunits modified by the lipid peroxidation product 4-hydroxy-2-nonenal. Biochemistry.

[101] Bennaars-Eiden A., Higgins L., Hertzel A.V., Kapphahn R.J., Ferrington D.A., Bernlohr D.A. (2002). Covalent modification of epithelial fatty acid-binding protein by 4-hydroxynonenal in vitro and in vivo. Evidence for a role in antioxidant biology. J. Biol. Chem..

[102] Sampey B.P., Carbone D.L., Doorn J.A., Drechsel D.A., Petersen D.R. (2007). 4-Hydroxy-2-nonenal adduction of extracellular signal-regulated kinase (Erk) and the inhibition of hepatocyte Erk-Est-like protein-1-activating protein-1 signal transduction. Mol. Pharmacol..

[103] Szweda L.I., Uchida K., Tsai L., Stadtman E.R. (1993). Inactivation of glucose-6-phosphate dehydrogenase by 4-hydroxy-2-nonenal. Selective modification of an active-site lysine. J. Biol. Chem..

[104] Backos D.S., Fritz K.S., Roede J.R., Petersen D.R., Franklin C.C. (2011). Posttranslational modification and regulation of glutamate-cysteine ligase by the α,β-unsaturated aldehyde 4-hydroxy-2-nonenal. Free Radic. Biol. Med..

[105] Mitchell A.E., Morin D., Lamé M.W., Jones A.D. (1995). Purification, Mass Spectrometric Characterization, and Covalent Modification of Murine Glutathione S-Transferases. Chem. Res. Toxicol..

[106] Ishii T., Tatsuda E., Kumazawa S., Nakayama T., Uchida K. (2003). Molecular basis of enzyme inactivation by an endogenous electrophile 4-hydroxy-2-nonenal: Identification of modification sites in glyceraldehyde-3-phosphate dehydrogenase. Biochemistry.

[107] Carbone D.L., Doorn J.A., Kiebler Z., Sampey B.P., Petersen D.R. (2004). Inhibition of Hsp72-mediated protein refolding by 4-hydroxy-2-nonenal. Chem. Res. Toxicol..

[108] Carbone D.L., Doorn J.A., Kiebler Z., Ickes B.R., Petersen D.R. (2005). Modification of heat shock protein 90 by 4-hydroxynonenal in a rat model of chronic alcoholic liver disease. J. Pharmacol. Exp. Ther..

[109] Aldini G., Gamberoni L., Orioli M., Beretta G., Regazzoni L., Maffei Facino R., Carini M. (2006). Mass spectrometric characterization of covalent modification of human serum albumin by 4-hydroxy-trans-2-nonenal. J. Mass Spectrom..

[110] Smathers R.L., Fritz K.S., Galligan J.J., Shearn C.T., Reigan P., Marks M.J., Petersen D.R. (2012). Characterization of 4-HNE modified L-FABP reveals alterations in structural and functional dynamics. PLoS ONE.

[111] Doorn J.A., Hurley T.D., Petersen D.R. (2006). Inhibition of human mitochondrial aldehyde dehydrogenase by 4-hydroxynon-2-enal and 4-oxonon-2-enal. Chem. Res. Toxicol..

[112] Roede J.R., Carbone D.L., Doorn J.A., Kirichenko O.V., Reigan P., Petersen D.R. (2008). In vitro and in silico characterization of peroxiredoxin 6 modified by 4-hydroxynonenal and 4-oxononenal. Chem. Res. Toxicol..

[113] Shearn C.T., Smathers R.L., Backos D.S., Reigan P., Orlicky D.J., Petersen D.R. (2013). Increased carbonylation of the lipid phosphatase PTEN contributes to Akt2 activation in a murine model of early alcohol-induced steatosis. Free Radic. Biol. Med..

[114] Camarillo J.M., Ullery J.C., Rose K.L., Marnett L.J. (2017). Electrophilic Modification of PKM2 by 4-Hydroxynonenal and 4-Oxononenal Results in Protein Cross-Linking and Kinase Inhibition. Chem. Res. Toxicol..

[115] Fritz K.S., Galligan J.J., Smathers R.L., Roede J.R., Shearn C.T., Reigan P., Petersen D.R. (2011). 4-hydroxynonenal inhibits SIRT3 via thiol-specific modification. Chem. Res. Toxicol..

[116] Jang E.J., Jeong H.O., Park D., Kim D.H., Choi Y.J., Chung K.W., Park M.H., Yu B.P., Chung H.Y. (2015). Src Tyrosine Kinase Activation by 4-Hydroxynonenal Upregulates p38, ERK/AP-1 Signaling and COX-2 Expression in YPEN-1 Cells. PLoS ONE.

[117] Stewart B.J., Doom J.A., Petersen D.R. (2007). Residue-specific adduction of tubulin by 4-hydroxynonenal and 4-oxononenal causes cross-linking and inhibits polymerization. Chem. Res. Toxicol..

[118] Dalleau S., Baradat M., Guéraud F., Huc L. (2013). Cell death and diseases related to oxidative stress:4-hydroxynonenal (HNE) in the balance. Cell Death Differ..

[119] Tang D., Chen X., Kang R., Kroemer G. (2021). Ferroptosis: Molecular mechanisms and health implications. Cell Res..

[120] Biasi F., Vizio B., Mascia C., Gaia E., Zarkovic N., Chiarpotto E., Leonarduzzi G., Poli G. (2006). c-Jun N-terminal kinase upregulation as a key event in the proapoptotic interaction between transforming growth factor-beta1 and 4-hydroxynonenal in colon mucosa. Free Radic. Biol. Med..

[121] Bauer G., Zarkovic N. (2015). Revealing mechanisms of selective, concentration-dependent potentials of 4-hydroxy-2-nonenal to induce apoptosis in cancer cells through inactivation of membrane-associated catalase. Free Radic. Biol. Med..

[122] Zhong H., Xiao M., Zarkovic K., Zhu M., Sa R., Lu J., Tao Y., Chen Q., Xia L., Cheng S. (2017). Mitochondrial control of apoptosis through modulation of cardiolipin oxidation in hepatocellular carcinoma: A novel link between oxidative stress and cancer. Free Radic. Biol. Med..

[123] Sunjic S.B., Gasparovic A.C., Jaganjac M., Rechberger G., Meinitzer A., Grune T., Kohlwein S.D., Mihaljevic B., Zarkovic N. (2021). Sensitivity of Osteosarcoma Cells to Concentration-Dependent Bioactivities of Lipid Peroxidation Product 4-Hydroxynonenal Depend on Their Level of Differentiation. Cells.

[124] Žarković N., Jaganjac M., Žarković K., Gęgotek A., Skrzydlewska E. (2022). Spontaneous Regression of Cancer: Revealing Granulocytes and Oxidative Stress as the Crucial Double-edge Sword. Front. Biosci. (Landmark Ed.).

[125] Shoeb M., Ansari N.H., Srivastava S.K., Ramana K. (2014). V 4-Hydroxynonenal in the pathogenesis and progression of human diseases. Curr. Med. Chem..

[126] Poll G., Leonarduzzi G., Charpotto E., Forman H.J., Fukuto J., Torres M. (2003). 4-Hydroxynonenal Signaling. Signal Transduction by Reactive Oxygen and Nitrogen Species: Pathways and Chemical Principles.

[127] Sonowal H., Ramana K. (2019). V 4-Hydroxy-Trans-2-Nonenal in the Regulation of Anti-Oxidative and Pro-Inflammatory Signaling Pathways. Oxid. Med. Cell. Longev..

[128] Sharma S., Sharma P., Bailey T., Bhattarai S., Subedi U., Miller C., Ara H., Kidambi S., Sun H., Panchatcharam M. (2022). Electrophilic Aldehyde 4-Hydroxy-2-Nonenal Mediated Signaling and Mitochondrial Dysfunction. Biomolecules.

[129] Shireman L.M., Kripps K.A., Balogh L.M., Conner K.P., Whittington D., Atkins W.M. (2010). Glutathione transferase A4-4 resists adduction by 4-hydroxynonenal. Arch. Biochem. Biophys..

[130] Yang Y., Huycke M.M., Herman T.S., Wang X. (2016). Glutathione S-transferase alpha 4 induction by activator protein 1 in colorectal cancer. Oncogene.

[131] Jaganjac M., Milkovic L., Sunjic S.B., Zarkovic N. (2020). The NRF2, Thioredoxin, and Glutathione System in Tumorigenesis and Anticancer Therapies. Antioxidants.

[132] Dinkova-Kostova A.T., Kostov R.V., Canning P. (2017). Keap1, the cysteine-based mammalian intracellular sensor for electrophiles and oxidants. Arch. Biochem. Biophys..

[133] Chen Z.-H., Saito Y., Yoshida Y., Sekine A., Noguchi N., Niki E. (2005). 4-Hydroxynonenal induces adaptive response and enhances PC12 cell tolerance primarily through induction of thioredoxin reductase 1 via activation of Nrf2. J. Biol. Chem..

[134] Fang X., Fu Y., Long M.J.C., Haegele J.A., Ge E.J., Parvez S., Aye Y. (2013). Temporally controlled targeting of 4-hydroxynonenal to specific proteins in living cells. J. Am. Chem. Soc..

[135] Gall Trošelj K., Tomljanović M., Jaganjac M., Matijević Glavan T., Čipak Gašparović A., Milković L., Borović Šunjić S., Buttari B., Profumo E., Saha S. (2022). Oxidative Stress and Cancer Heterogeneity Orchestrate NRF2 Roles Relevant for Therapy Response. Molecules.

[136] Malhotra D., Portales-Casamar E., Singh A., Srivastava S., Arenillas D., Happel C., Shyr C., Wakabayashi N., Kensler T.W., Wasserman W.W. (2010). Global mapping of binding sites for Nrf2 identifies novel targets in cell survival response through ChIP-Seq profiling and network analysis. Nucleic Acids Res..

[137] Gao Q., Zhang G., Zheng Y., Yang Y., Chen C., Xia J., Liang L., Lei C., Hu Y., Cai X. (2020). SLC27A5 deficiency activates NRF2/TXNRD1 pathway by increased lipid peroxidation in HCC. Cell Death Differ..

[138] Hong F., Freeman M.L., Liebler D.C. (2005). Identification of sensor cysteines in human Keap1 modified by the cancer chemopreventive agent sulforaphane. Chem. Res. Toxicol..

[139] McMahon M., Lamont D.J., Beattie K.A., Hayes J.D. (2010). Keap1 perceives stress via three sensors for the endogenous signaling molecules nitric oxide, zinc, and alkenals. Proc. Natl. Acad. Sci. USA.

[140] Parvez S., Fu Y., Li J., Long M.J.C., Lin H.-Y., Lee D.K., Hu G.S., Aye Y. (2015). Substoichiometric hydroxynonenylation of a single protein recapitulates whole-cell-stimulated antioxidant response. J. Am. Chem. Soc..

[141] Lu B., Chen X.B., Ying M.D., He Q.J., Cao J., Yang B. (2017). The Role of Ferroptosis in Cancer Development and Treatment Response. Front. Pharmacol..

[142] Chen X., Huang J., Yu C., Liu J., Gao W., Li J., Song X., Zhou Z., Li C., Xie Y. (2022). A noncanonical function of EIF4E limits ALDH1B1 activity and increases susceptibility to ferroptosis. Nat. Commun..

[143] Chen Y., Liu Y., Lan T., Qin W., Zhu Y., Qin K., Gao J., Wang H., Hou X., Chen N. (2018). Quantitative Profiling of Protein Carbonylations in Ferroptosis by an Aniline-Derived Probe. J. Am. Chem. Soc..

[144] Yang Y., Luo M., Zhang K., Zhang J., Gao T., Connell D.O., Yao F., Mu C., Cai B., Shang Y. (2020). Nedd4 ubiquitylates VDAC2/3 to suppress erastin-induced ferroptosis in melanoma. Nat. Commun..

[145] Brown C.W., Chhoy P., Mukhopadhyay D., Karner E.R., Mercurio A.M. (2021). Targeting prominin2 transcription to overcome ferroptosis resistance in cancer. EMBO Mol. Med..

[146] Jacobs A.T., Marnett L.J. (2007). Heat Shock Factor 1 Attenuates 4-Hydroxynonenal-mediated Apoptosis. J. Biol. Chem..

[147] Cyran A.M., Zhitkovich A. (2022). Heat Shock Proteins and HSF1 in Cancer. Front. Oncol..

